# Signatures of Mollicutes-related endobacteria in publicly available Mucoromycota genomes

**DOI:** 10.1128/msphere.00309-24

**Published:** 2024-08-27

**Authors:** Reid Longley, Aaron J. Robinson, Olivia A. Asher, Earl Middlebrook, Gregory Bonito, Patrick S. G. Chain

**Affiliations:** 1 Department of Plant, Soil and Microbial Sciences, Michigan State University, East Lansing, Michigan, USA; 2 Department of Microbiology and Molecular Genetics, Michigan State University, East Lansing, Michigan, USA; 3 Los Alamos National Laboratory, Los Alamos, New Mexico, USA; 4 Institute of Bioinformatics, University of Georgia, Athens, Georgia, USA; University College Dublin, Dublin, Ireland

**Keywords:** bacterial–fungal interactions, intermicrobial, genomics, bioinformatics, mucormycota, endobacteria, Mollicutes

## Abstract

Mucoromycota fungi and their Mollicutes-related endobacteria (MRE) are an ideal system for studying bacterial–fungal interactions and evolution due to the long-term and intimate nature of their interactions. However, methods for detecting MRE face specific challenges due to the poor representation of MRE in sequencing databases coupled with the high sequence divergence of their genomes, making traditional similarity searches unreliable. This has precluded estimations on the diversity of MRE associated with Mucoromycota. To determine the prevalence of previously undetected MRE in fungal genome sequences, we scanned 389 Mucoromycota genome assemblies available from the National Center for Biotechnology Information for the presence of MRE sequences using publicly available tools to map contigs from fungal assemblies to publicly available MRE genomes. We demonstrate a higher diversity of MRE genomes than previously described in Mucoromycota and a lack of cophylogeny between MRE and the majority of their fungal hosts. This supports the late invasion hypothesis regarding MRE acquisition across most of the examined fungal families. In contrast with other Mucoromycota lineages, MRE from the Gigasporaceae displayed some degree of cophylogeny with their hosts, which may indicate that horizontal transmission is restricted between members of this family or that transmission is strictly vertical. These results underscore the need for a refined process to capture sequencing data from potential fungal endosymbionts to discern their evolution and transmission. Screens of fungal genomes for MRE can help improve the quality of fungal genome assemblies while identifying new MRE lineages to further test hypotheses on their origin and evolution.

**IMPORTANCE:**

Mollicutes-related endobacteria (MRE) are obligate intracellular bacteria found within Mucoromycota fungi. Despite their frequent detection, MRE roles in host functioning are still unknown. Comparative genomic investigations can improve our understanding of the impact of MRE on their fungal hosts by identifying similarities and differences in MRE genome evolution. However, MRE genomes have only been assembled from a small fraction of Mucoromycota hosts. Here, we demonstrate that MRE can be present yet undetected in publicly available Mucoromycota genome assemblies. We use these newfound sequences to assess the broader diversity of MRE and their phylogenetic relationships with respect to their hosts. We demonstrate that publicly available tools can be used to extract novel MRE sequences from assembled fungal genomes leading to insights on MRE evolution. This work contributes to a greater understanding of the fungal microbiome, which is crucial to improving knowledge on the dynamics and impacts of fungi in microbial ecosystems.

## OBSERVATION

Mucoromycota fungi are known to host two types of obligate intracellular endobacteria, *Burkholderia*-related endobacteria (BRE) and Candidatus Moeniiplasma known as Mollicutes-related endobacteria (MRE) (1). BRE have been shown to impact host biology and pathogenicity, but the roles of MRE in host functioning or interactions with other organisms remain largely unknown (2, 3). Genome-based investigations revealed that MRE have a complex evolutionary history. Their lack of cophylogeny with fungal hosts is indicative of host-switching and an invasion into Mucoromycota fungi after the divergence of fungal hosts from a common ancestor (4, 5). However, these inferences are based on merely 14 publicly available MRE genomes despite the 389 Mucoromycota fungal genome assemblies present in GenBank. Due to the high degree of sequence divergence between MRE lineages and a lack of representation in databases, detecting MRE in sequencing data is expected to be difficult. Undetected endobacteria sequences could lead to spurious inclusion of bacterial sequences in fungal genome assemblies. To evaluate the possibility and extent of this phenomenon, we downloaded all publicly available Mucoromycota genomes from GenBank and screened for the presence of MRE using publicly available tools.

Contigs from fungal genome assemblies were mapped using Minimap2 (6) to a local database containing 13 MRE genome assemblies. Contigs that mapped to MRE assemblies with perfect alignment scores (60) were considered to be of putative MRE origin. These MRE contigs were then further assessed using National Center for Biotechnology Information (NCBI) BLAST. Contigs that aligned to sequences (*e* < −10) from Mollicutes, MRE, or had no hits in the BLAST nucleotide database (nr/nt) were retained (7). In addition to reference-based analysis, VizBin was used as a non-reference-based method to identify contigs that clustered separately from fungal contigs (8). Contigs identified with VizBin that aligned to Mollicutes with NCBI BLAST (*e* < −10) were added to putative MRE assemblies. Because a single fungal host may contain multiple MRE phylotypes, MRE contigs were binned with MaxBin2 to separate multiple MRE phylotypes present in a single fungal genome assembly (9–11). In some cases, multiple rounds of successive binning were used to identify and separate contigs from distinct MRE lineages. MaxBin2 was also utilized to recover two additional MRE from a previously published Endogonales MRE meta-assembly, which was known to contain multiple MRE phylotypes (10) (Table S1). MRE assemblies were then assessed for completeness and contamination (i.e., duplications of single-copy genes) with CheckM using a reference set of single-copy genes from Mollicutes, and bins identified as Mollicutes were assessed further (12). Fungal genome assemblies containing putative MRE contigs were also assessed with NCBI’s foreign contamination screen tool (13). A phylogenetic analysis of previously and newly identified MRE sequences was performed using the OrthoPhyl phylogenetic pipeline (14). Details on phylogenetics and further assessments of MRE genome assemblies are described in the supplementary methods. Additionally, to assess the effectiveness of Minimap2 for detecting other bacteria, Mucoromycota genomes were scanned for the presence of BRE using Minimap2 against a database of 14 BRE genomes followed by BLAST filtering as described above.

We recovered 20 putative MRE assemblies previously unidentified from 14 fungal host genomes and two additional Endogonales MRE genomes from a previously published MRE meta-assembly. Five additional fungal genomes contained smaller fragments of MRE genomes (9–130 kb), which were not included in further analysis given their limited size (Table S1). Our results may underestimate the incidence of chimeric MRE sequences in Mucoromycota genomes due to the small number of publicly available MRE genomes available to map against fungal genomes. In some cases, mapping with Minimap2 resulted in false-positive detections, possibly due to horizontal gene transfer of fungal genes into MRE genomes or contamination of MRE assemblies in the database with fungal host contigs. False positives were readily identified and filtered as these sequences only had BLAST hits to fungal genomes. Additionally, bins that were not identified as Mollicutes by CheckM were not assessed further but may contain MRE sequences that could not be confidently binned. This may indicate the presence of additional MRE phylotypes or other bacteria in cases where binning substantially reduced the assembly size. In each case, the results were supported by the NCBI foreign contamination screen tool that also detected the presence of MRE, but in some cases, missed contigs that were recovered by Minimap2 (Table S1). Extracted MRE genomes varied in completeness, but CheckM revealed that several were of comparable completeness (>45%) to complete MRE genome assemblies (4). Recovered MRE genome assemblies ranged in size from 314,771 to 970,273 bp and had GC contents ranging from 29% to 33%. Similar to previously sequenced MRE and other endosymbionts, assemblies were generally enriched in pseudogenes, repeats, and phage content (Table S1; Fig. S1) (4, 10, 15–18). Extracted MRE genomes were generally enriched in *XerC*/*XerD* site-specific recombinases that support the importance of genome rearrangement in MRE evolution (Fig. S1) (4, 19). Additionally, Minimap2 and BLAST indicated the presence of BRE sequences in six Mucoromycota genomes indicating that these methods should be effective in detecting additional groups of bacteria. However, unlike MRE, detected sequences were primarily from assemblies where BRE were previously detected, indicating that these sequences were unlikely to be complete genomes but instead contigs that were missed by other tools. Assemblies containing putative BRE contigs are shown in Table S2.

**Fig 1:**
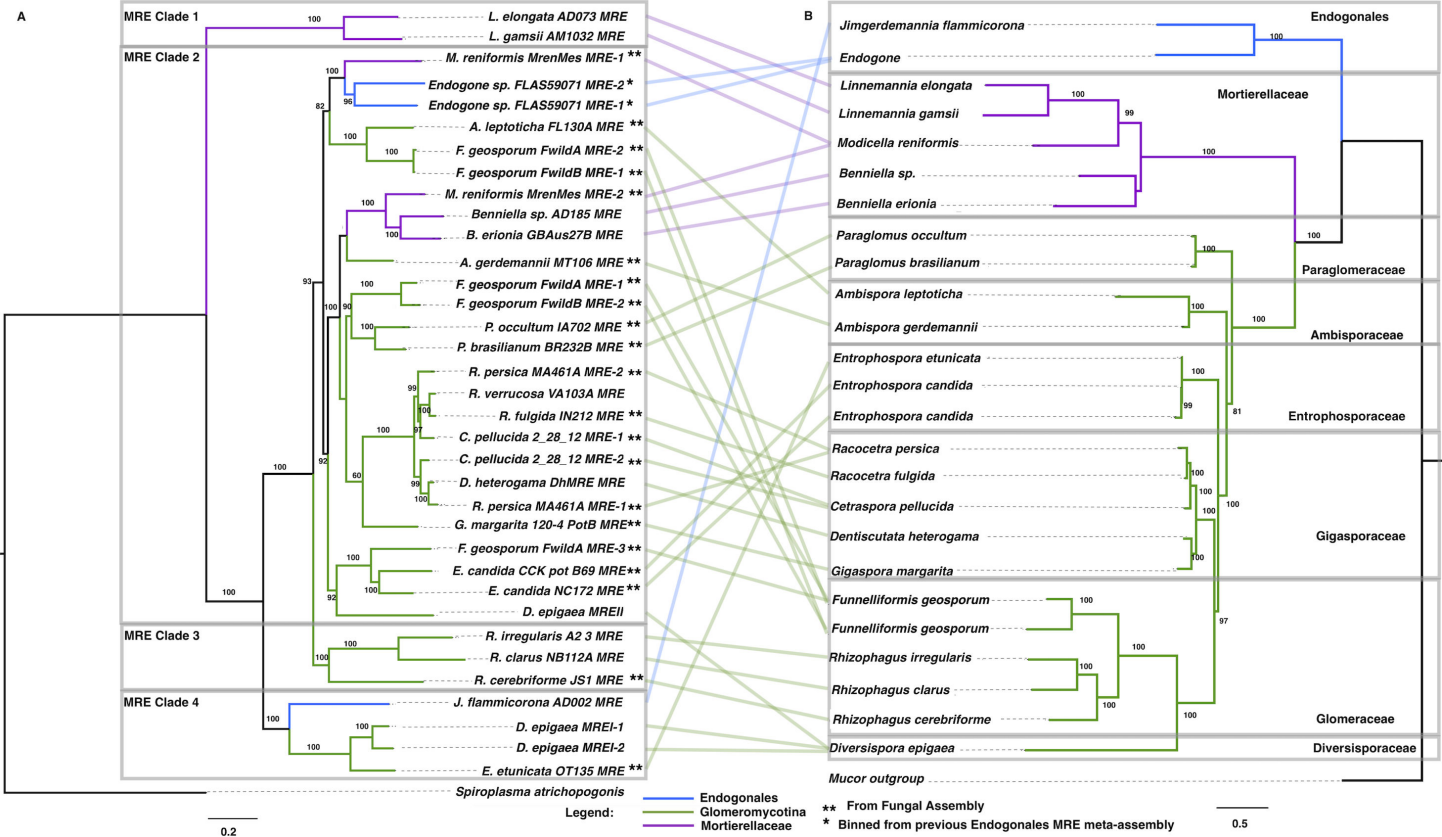
Phylogenetics provides further support for the late invasion hypothesis due to broad lack of cophylogeny between MRE and fungal hosts. (A) Maximum likelihood phylogeny of MRE extracted from fungal assemblies and previously published MRE genome assemblies. (**B**) Maximum likelihood phylogeny of fungal hosts of MRE created using 22 shared BUSCO genes. (Table S3). ** represents novel MRE assemblies identified from fungal genome assemblies. * represents MRE assemblies binned from publicly available Endogonales MRE meta-assembly. Lines connect MRE taxa to their fungal host species. Branch labels indicate bootstrap values (1,000 replicates). Bootstrap values > 50 are shown.

The majority of MRE genomes identified in this screen appear to be related to lineages assigned to the previously designated MRE clade 2 (Fig. 1). Fungal phylogenies created using alignments of 22 BUSCO genes clustered taxa as expected with distinct separation of fungal families. Fungal genome sources and BUSCO genes used to create phylogenies are shown in Table S3 and S4. The additional mined MRE genomes further support the late invasion hypothesis of MRE evolution, given the overall lack of cophylogeny with their fungal hosts (4, 5). For example, MRE from Glomeraceae lacked cophylogeny with their fungal hosts (Fig. 1). However, representatives from each of the Gigasporaceae and the Paraglomeraceae were clustered phylogenetically by fungal host family. This may indicate strictly vertical MRE transmission in these hosts or that horizontal transmission of MRE only occurs between confamilial hosts. Gigasporaceae taxa are known to differ from the Glomeraceae due to their inability for anastomosis between distinct hyphal networks of the same isolate and are therefore unlikely to be able to form large hyphal networks between multiple plants (20). This may reduce the opportunity for lateral cytoplasmic transfer of MRE. These hypotheses remain to be tested experimentally and validated by further sampling.

**Fig 2:**
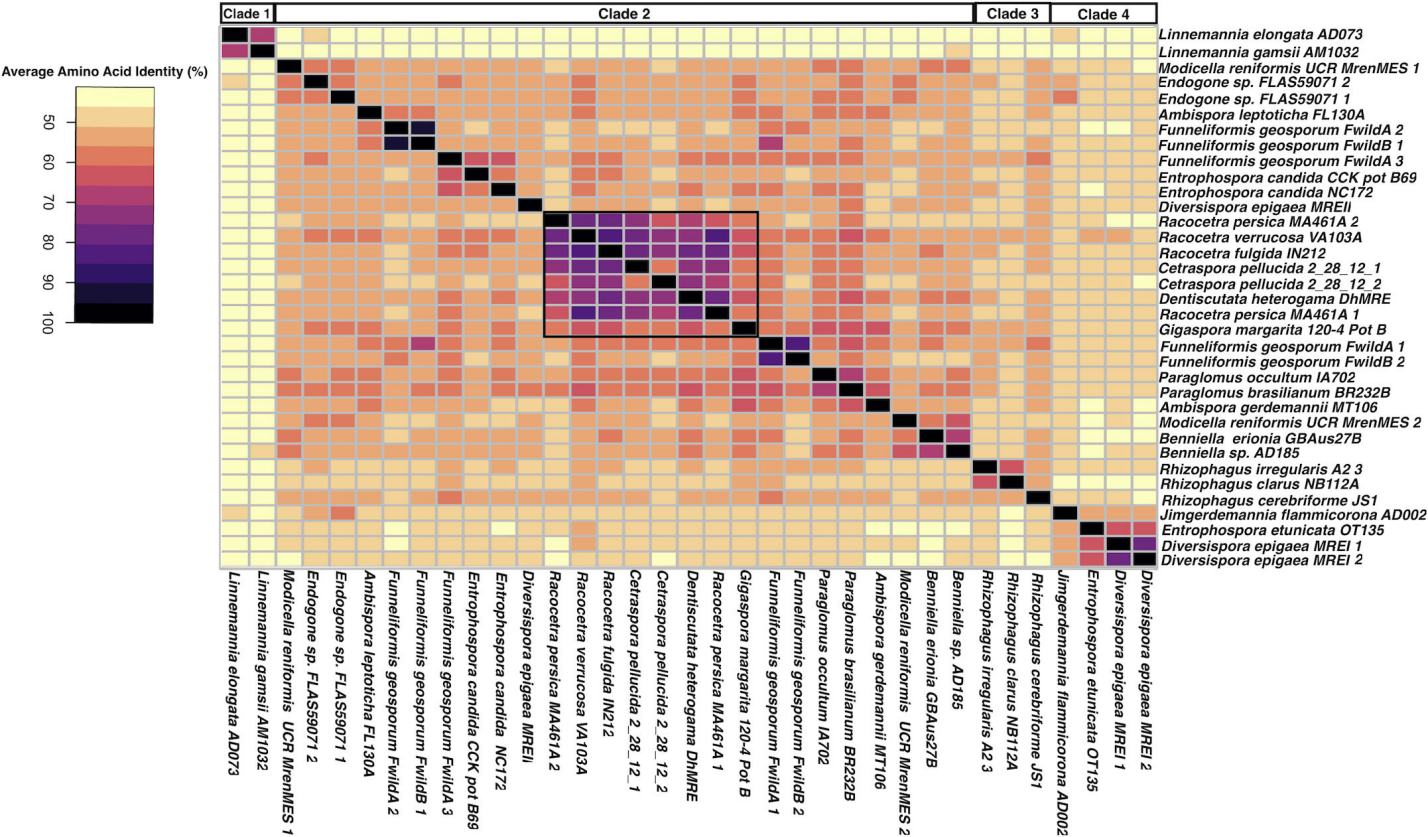
Heatmap showing pairwise AAI between MRE taxa. AAI is below 70% between most MRE taxa, but MRE from Gigasporaceae hosts generally display higher AAI. Black rectangle indicates MRE from Gigasporaceae hosts.

Pairwise average nucleotide identity (ANI) and average amino acid identity (AAI) were calculated as described in the supplementary methods. The majority of ANI comparisons between MRE were below a 70% cutoff utilized by the *enveomics* toolkit as below this threshold, differences in divergence are difficult to assess (Fig. S2; Table S5) (21). MRE from the Gigasporaceae had higher pairwise ANI although most of these pairwise comparisons fell below the 94% ANI threshold that is inferred to indicate strains of the same species (22). As the majority of MRE were too divergent from each other to reliably compare with ANI, AAI was also calculated to assess genomic divergence. AAI metrics agreed with ANI, indicating increased similarity between Gigasporaceae MRE. Between Gigasporaceae MRE, pairwise AAI values were generally above 70%, while pairwise values between other MRE taxa were near or below 60% (Fig. 2; Table S5). Together, ANI and AAI indicate that MRE from the Gigasporaceae are more closely related to each other compared to increased divergence between MRE from other host groups. However, Glomeraceae representatives *Funneliformis geosporum* FwildA and FwildB, which each host has multiple MRE, hosted similar MRE between the two host strains. The lower divergence among MRE taxa from the Gigasporaceae may indicate a more recent invasion, strict vertical transmission, or restricted horizontal transmission. There is no standard AAI threshold for delineating taxonomic groups, but thresholds generally vary between 60% and 80% to separate genera (23, 24). These thresholds may be arbitrary and depend on the taxonomic group due to differences in sampling and rates of evolution. Any threshold to distinguish Mollicutes genera would be expected to be lower due to an expected high rate of evolution (25).

In summary, publicly available tools were used to reveal chimeric MRE–Mucoromycota genome assemblies. MRE assemblies extracted and characterized herein provide further support for the late invasion hypothesis. Our results indicate that horizontal transmission of MRE may vary by host lineage. Given the detection of MRE in 19 out of 389 fungal genome assemblies (~5%), we recommend the use of techniques described here to ensure the detection and assembly of MRE genomes, which will allow researchers to address hypotheses regarding MRE evolution more effectively.

## SUPPLEMENTAL MATERIAL

Figure S1 msphere.00309-24-s0001.tiff Percentage of each genome composed of pseudogenes, repeats, and phage content and copy numbers of XerC and XerD site-specific recombinases in MRE genomes.

Figure S2 msphere.00309-24-s0002.tiff Heatmap showing pairwise ANI distances between MRE taxa.

Supplemental Methods msphere.00309-24-s0003.docx Detailed phylogenetic methods and MRE annotation details.

Legends msphere.00309-24-s0004.docx Descriptions of supplemental material files.

Table S1 msphere.00309-24-s0005.csv Summary statistics of MRE genomes extracted from fungal assemblies and previously published MRE assemblies.

Table S2 msphere.00309-24-s0006.csv Fungal assemblies that contained putative BRE contigs.

Table S3 msphere.00309-24-s0007.csv Sources for fungal genomes used in phylogeny in Fig. 1B.

Table S4 msphere.00309-24-s0008.csv BUSCO genes for fungal phylogeny in Fig. 1B.

Table S5 msphere.00309-24-s0009.csv Raw values for pairwise average amino acid identities and average nucleotide identities shown in Fig. 2 and S2.

ASM does not own the copyrights to Supplemental Material that may be linked to, or accessed through, an article. The authors have granted ASM a non-exclusive, world-wide license to publish the Supplemental Material files. Please contact the corresponding author directly for reuse.
