# Erratum for Longley et al., “Signatures of Mollicutes-related endobacteria in publicly available Mucoromycota genomes”

**DOI:** 10.1128/msphere.00881-24

**Published:** 2024-11-18

**Authors:** Reid Longley, Aaron J. Robinson, Olivia A. Asher, Earl Middlebrook, Gregory Bonito, Patrick S. G. Chain

**Affiliations:** 1 Los Alamos National Laboratory, Los Alamos, New Mexico, USA; 2 Institute of Bioinformatics, University of Georgia, Athens, Georgia, USA; 3 Department of Plant, Soil and Microbial Sciences, Michigan State University, East Lansing, Michigan, USA; 4 Department of Microbiology and Molecular Genetics, Michigan State University, East Lansing, Michigan, USA

## ERRATUM

Volume 9, no. 9, e00309-24, 2024, https://doi.org/10.1128/msphere.00309-24. The affiliation line and the affiliation letters for each author should appear as shown in this erratum.

