# Supplementary figures and images for: Signatures of Mollicutes-related endobacteria in publicly available Mucoromycota genomes
Source: mSphere. 2024 Aug 27;9(9):e00309-24. doi: 10.1128/msphere.00309-24 (PMC11423566; doi:10.1128/msphere.00309-24)

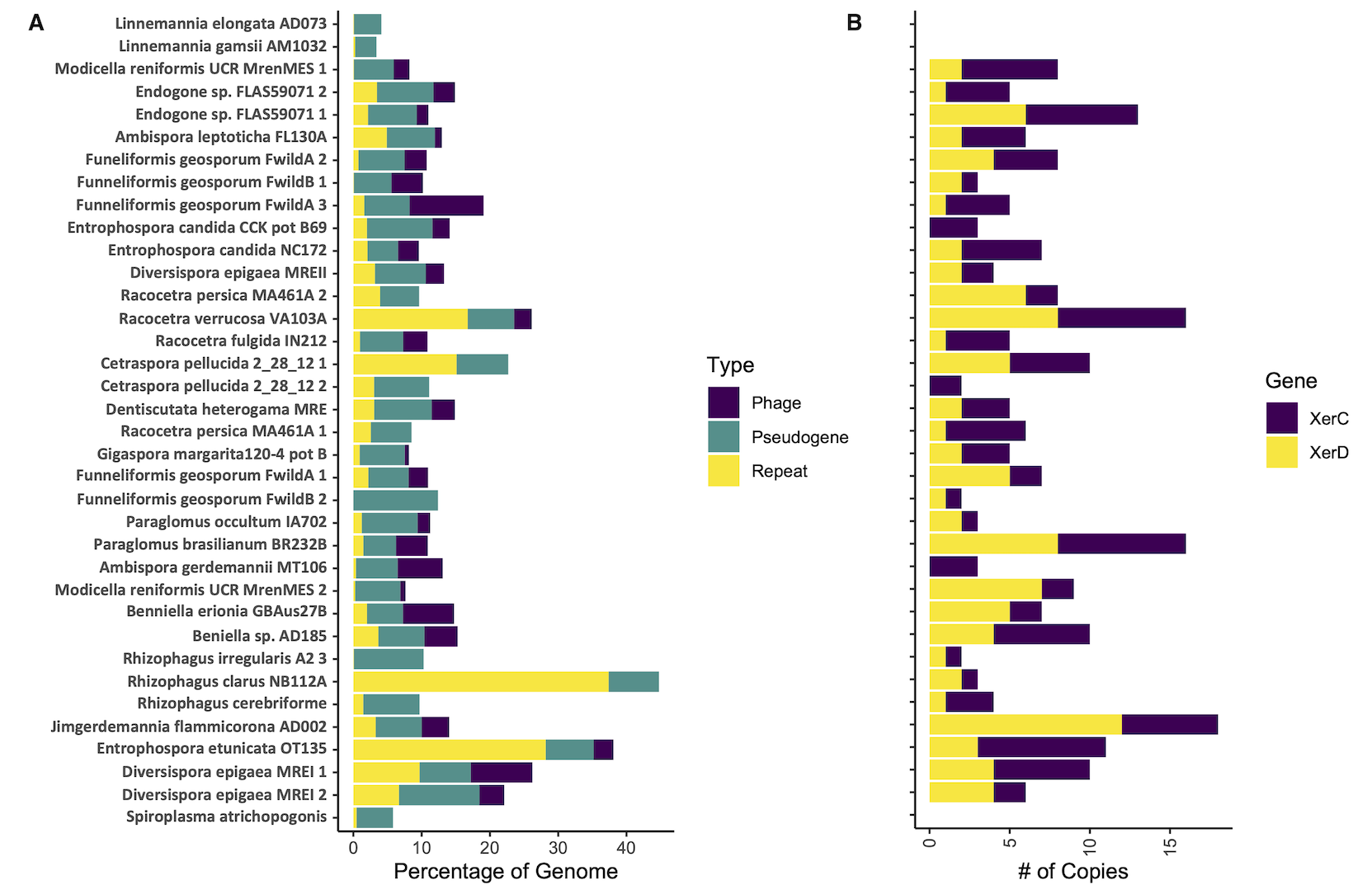

Supplement: Figure S1 — Percentage of each genome composed of pseudogenes, repeats, and phage content and copy numbers of XerC and XerD site-specific recombinases in MRE genomes. [file msphere.00309-24-s0001.tiff]

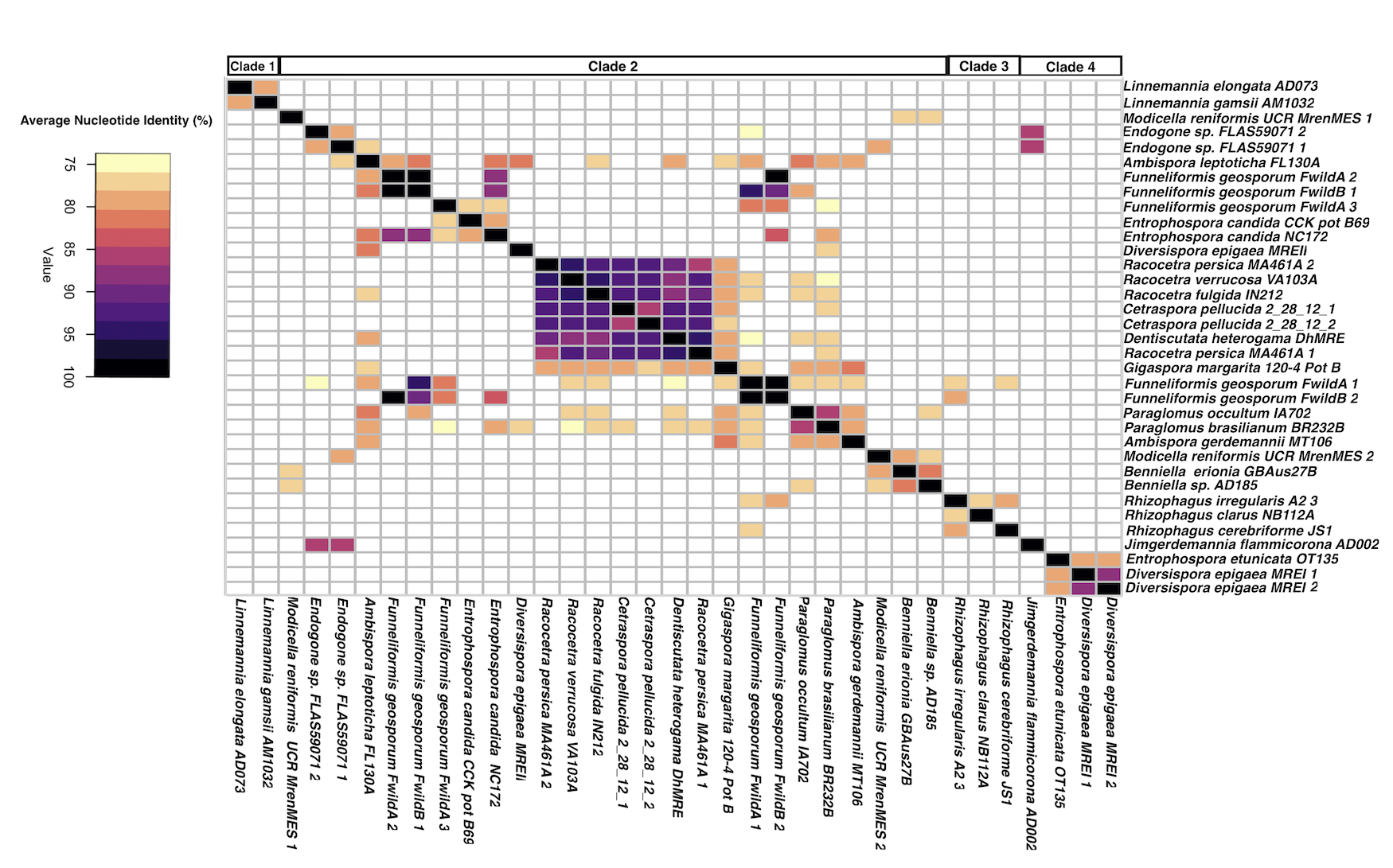

Supplement: Figure S2 — Heatmap showing pairwise ANI distances between MRE taxa. [file msphere.00309-24-s0002.tiff]
