# Supplementary material for: Signatures of Mollicutes-related endobacteria in publicly available Mucoromycota genomes
Source: mSphere. 2024 Aug 27;9(9):e00309-24. doi: 10.1128/msphere.00309-24 (PMC11423566; doi:10.1128/msphere.00309-24)
Supplement: Supplemental Methods — Detailed phylogenetic methods and MRE annotation details. [file msphere.00309-24-s0003.docx]

**Supplementary Methods:**

MRE phylogenies were created using the OrthoPhyl pipeline (1). Briefly, this pipeline took raw MRE genome assemblies extracted from fungal host genome assemblies as input. The assemblies were annotated using Prodigal with a Mollicutes genetic code (Translation Table 4) (2). Ortholog groups were then identified using Orthofinder (3). To maximize the number of genes utilized in MRE phylogenies, single copy orthologs found in at least 30% of assemblies were retained. Next, these single copy ortholog protein sequences were aligned, then used to produce nucleotide alignments of CDS sequences. Sequences were trimmed with *trimal* with *“-resoverlap .5 -seqoverlap 50 -gt .80 -cons 60 -w 3”* set as parameters (4). Finally, these gene alignments were concatenated into a single supermatrix of 44.2kb length using catfast2phyml (<https://github.com/nylander/catfasta2phyml>). Phylogenies were inferred from the supermatrix with RAxML under the GTR + GAMMA rate model and 100 bootstrap replicates were generated to calculate split support. Shared single copy orthologs were identified from fungal hosts with BUSCO via the gVolante web interface (5, 6). Phylogenies were then inferred using RAxML with automatic model selection of the best protein substitution model (-m PROTGAMMAAUTO) and 1000 bootstrap replicates (7). All internal programs were run with default parameters unless otherwise specified.

Following phylogenetic analysis, novel MRE genomes extracted from fungal assemblies were annotated with the Mollicutes genetic code (Translation Table 4) using Prokka with an e value threshold of 0.01 (8). Pseudogenes were annotated using the *pseudofinder* tool with default parameters and repeats were identified from RASTtk annotations. Phage content was identified with PhiSpy using default parameters (9,10). MRE and host phylogenies were visualized using FigTree V1.4.4 (<http://tree.bio.ed.ac.uk/software/figtree/>). Divergence between MRE taxa was also assessed using average pairwise average nucleic identity (ANI) and amino acid identity (AAI) using the all vs all ANI/AAI matrix calculator in the *enveomics* collection (11,12). ANI was calculated using default parameters (minimum alignment length of 700 bp, minimum identity of 70%, and a minimum of 50 alignments). AAI was calculated on shared proteins annotated with Prokka using the AAI calculator with default parameters (minimum identity of 20% and a minimum of 50 alignments) in the *enveomics* collection (8, 11,12). Heatmaps were created using the “heatmap.2” function in the *gplots* package of R (13). Other visualizations were created with R studio using the *ggplot2* package (14).

**References:**

1. Middlebrook EA, Katani R, Fair JM. 2024. OrthoPhyl – Streamlining large scale, orthology-based phylogenomic studies of bacteria at broad evolutionary scales. G3: Genes Genom Genet jkae119.

2. Hyatt D, Chen G-L, Locascio PF, Land ML, Larimer FW, Hauser LJ. 2010. Prodigal: prokaryotic gene recognition and translation initiation site identification. BMC Bioinformatics 11:119.

3. Emms DM, Kelly S. 2019. OrthoFinder: phylogenetic orthology inference for comparative genomics. Genome Biol 20:238.

4. Capella-Gutiérrez S, Silla-Martinez JM, Gabaldón T. 2009. trimAl: a tool for automated alignment trimming in large-scale phylogenetic analyses. Bioinformatics 25:1972–1973.

5. Nishimura O, Hara Y, Kuraku S. 2019. Evaluating genome assemblies and gene models using gVolante. Methods Mol Biol 1962:247–256.

6. Manni M, Berkeley MR, Seppey M, Simão FA, Zdobnov EM. 2021. BUSCO update: Novel and streamlined workflows along with broader and deeper phylogenetic coverage for scoring of eukaryotic, prokaryotic, and viral genomes. Mol Biol Evol 38:4647–4654.

7. Stamatakis A. 2014. RAxML version 8: a tool for phylogenetic analysis and post-analysis of large phylogenies. Bioinformatics 30:1312–1313.

8. Seemann T. 2014. Prokka: rapid prokaryotic genome annotation. Bioinformatics 30:2068–2069.

9. Syberg-Olsen MJ, Garber AI, Keeling PJ, McCutcheon JP, Husnik F. 2022. Pseudofinder: Detection of Pseudogenes in Prokaryotic Genomes. Mol Biol Evol 39: msac153.

10. Akhter S, Aziz RK, Edwards RA. 2012. PhiSpy: a novel algorithm for finding prophages in bacterial genomes that combines similarity- and composition-based strategies. Nucleic Acids Res 40: e126.

11. Rodriguez-R LM, Konstantinidis KT. 2016. The enveomics collection: a toolbox for specialized analyses of microbial genomes and metagenomes. Peerj Preprints 4:e1900v1.

12. Rodriguez-R LM, Konstantinidis KT. 2014. Bypassing Cultivation to Identify Bacterial Species. Microbe Magazine 9:111–118.

13. Warnes GR, Bolker B, Bonebakker L, Gentleman R, Huber W, Liaw A, Lumley T. 2009. gplots: Various R programming tools for plotting data. R package version 2.

14. Wickham H. 2009. ggplot2: elegant graphics for data analysis Springer-Verlag. Springer-Verlag, New York.
