# Supplementary material for: Signatures of Mollicutes-related endobacteria in publicly available Mucoromycota genomes
Source: mSphere. 2024 Aug 27;9(9):e00309-24. doi: 10.1128/msphere.00309-24 (PMC11423566; doi:10.1128/msphere.00309-24)
Supplement: Legends — Descriptions of supplemental material files. [file msphere.00309-24-s0004.docx]

**Supplementary Figure 1: A)** Percentage of each genome composed of pseudogenes, repeats, and phage content. **B)** Copy numbers of *XerC* and *XerD* site-specific recombinases in MRE genomes.

**Supplementary Figure 2:** Heatmap showing pairwise Average Nucleotide Identity (ANI) distances between MRE taxa. Black rectangle indicates MRE from Gigasporaceae hosts. Values below 70% are not shown.

**Supplementary Table 1:** Summary statistics of MRE genomes extracted from fungal assemblies and previously published MRE assemblies.

**Supplementary Table 2:** Fungal assemblies which contained putative BRE contigs.

**Supplementary Table 3:** Sources for fungal genomes used in phylogeny in Figure 1B.

**Supplementary Table 4:** BUSCO genes for fungal phylogeny in Figure 1B.

**Supplementary Table 5**: Raw values for pairwise average amino acid identities and average nucleotide identities shown in Figure 2 and Supplementary Figure 2.

**Supplementary Methods:** Details on MRE phylogenetics and annotations.
